# Physical rehabilitation in Iran after international sanctions: explored findings from a qualitative study

**DOI:** 10.1186/s12992-020-00618-8

**Published:** 2020-09-23

**Authors:** Saeed Shahabi, Ahmad Ahmadi Teymourlouy, Hosein Shabaninejad, Mohammad Kamali, Kamran Bagheri Lankarani, Parviz Mojgani

**Affiliations:** 1 Health Policy Research Center, Institute of Health, Shiraz University of Medical Sciences, Shiraz, Iran; 2 Department of Health Services Management, School of Health Management and Information Sciences, Iran University of Medical Sciences, Tehran, Iran; 3 Population Health Sciences Institute, Newcastle University, Newcastle, UK; 4 Rehabilitation Research Center, Department of Rehabilitation Management, School of Rehabilitation Sciences, Iran University of Medical Sciences, Tehran, Iran; 5 Iran-Helal Institute of Applied Science and Technology, Tehran, Iran

**Keywords:** Sanctions, Embargoes, Physical rehabilitation, Health policy, Qualitative study

## Abstract

**Background:**

Although the main aims of sanctions are the political and economic pressures on governments, literature has demonstrated the harsh effects of sanctions on the general public, especially on the patients, poor and disabled people. Since the international sanctions regime negatively affected almost all dimensions of Iran’s health sector, this qualitative study was conducted to investigate the situation of the physical rehabilitation sector after these sanctions.

**Methods:**

This qualitative study was conducted from January 2019 to June 2019 in Iran using Skype, telephone, and face-to-face in-depth semi-structured interviews. Purposive and snowball sampling approaches were used to identify the participants. Also, framework analysis approach was applied to analyze the collected data.

**Results:**

In total, 38 individuals including health policy-maker, faculty member, rehabilitation expert, Physiotherapist, Occupational therapist, and Orthotist/Prosthetist, were involved in the study. Based on our findings, a number of challenges facing the Iranian physical rehabilitation sector during the international sanctions period included: 1) socioeconomic challenges (inadequate funding, rising inflation rate, high unemployment rate, catastrophic expenditures, and inappropriate employment status of practitioners); 2) education challenges (decreased international collaboration and shortage of training devices and materials); 3) international challenges (rising issues in accessing services for patients from neighborhood countries); and 4) service delivery challenges (shortage of raw materials for producing the orthoses and prostheses, hardening of the importing the needed equipment, inappropriate infrastructures, and impossibility to use external assistance).

**Conclusion:**

After international sanctions, the Iranian physical rehabilitation sector has faced considerable multifaceted challenges. Therefore, the international community must be aware of the situation and be concerned about the irreparable consequences.

## Introduction

Sanctions and embargoes are designated as powerful political instruments to affect the economic activities of the targeted nation to force a change in the state’s behaviors and policies [1]. Following Iran’s Islamic Revolution and holding hostage of the U.S. diplomats at the U.S. embassy in Tehran in 1979, the former U.S. President ordered the initial sanctions against Iran [2]. Since then, several sanction regimes were imposed, especially during 2006–2010, in which multilateral sanctions were considered with the United Nations Security Council and Council of the European Union [3]. In response to this situation, Iran and the P5 + 1 (the five permanent members of the United Nations Security Council plus Germany) had intensive negotiations to lifting Iran’s nuclear sanctions, and finally, an agreement called the Joint Comprehensive Plan of Action (JCPOA) was established in July 2015. However, the newly elected U.S. President repealed the agreement and reimposed the U.S. sanctions against Iran 3 years later in November 2018 [4]. In fact, given that the main focus of these sanctions has been to reduce Iranian oil revenues and restrict the international activities of the Central Bank of Iran, such limited cooperation with other countries has had significant effects on various social and economic dimensions of Iran [5].

Although the main aims of sanctions are the political and economic pressures, previous literature has shown the harsh effects of sanctions on the general public, especially on the patients, poor and disabled people, with direct and indirect impacts on the health and well-being systems [6–8]. Following the aforementioned sanctions, Iran has confronted with various economic challenges in recent years such as reducing the oil exports and related revenues, banning the banks from international transactions, and restricting the imports including medicines and medical equipment, as well as devaluating the national currency and jumping the inflation rate. As a result, the sanction regime negatively affected almost all dimensions of Iran’s heath sector, including physical rehabilitation services [9, 10].

A major proportion of patients using physical rehabilitation services (including physiotherapy, occupational therapy, Orthotics and Prosthetics) are categorized as vulnerable groups that the sanctions and the ad hoc economic recession may have significant adverse effects on their utilization [11]. For instance, reports have demonstrated the negative impacts of Greece’s economic recession on the accessibility of treatment for multiple sclerosis and rheumatoid arthritis patients [12]. Therefore, monitoring and measuring the effects of sanctions and embargos on the physical rehabilitation sector and its targeted population can be a crucial strategy to develop comprehensive policies in order to diminish the current burdens.

In Iran, population aging, high rate of traffic injuries (amputations, fractures, and head trauma), chronic diseases, congenital disorders, natural disasters, and also climate changes, have increased the demand for physical rehabilitation services significantly [13, 14]. Nonetheless, previous studies reported several barriers, including financial hardships and shortage of materials as the results of international sanctions [7, 9], which might prevent equitable utilization. Therefore, based on the United Nations (UN) and the World Health Organization (WHO) guidelines and instructions, strengthening the physical rehabilitation in health systems and also facilitating the availability and accessibility of these services for the targeted population, especially disabled people, should be considered through the decision- and policy-making processes, both nationally and internationally [15–17].

To the best knowledge, although several studies have been conducted regarding the impacts of international sanctions on different parts of the Iranian health sector [1–3, 6, 18–23], there is no study that has evaluated the potential impacts of sanctions on physical rehabilitation functions in Iran.

In response, this qualitative study was conducted to investigate the situation of the physical rehabilitation sector in Iran after the international sanctions. The findings could also be used by international organizations to diminish the adverse effects of embargos on vulnerable populations such as disabled.

## Methods

This qualitative study was conducted from January 2019 to June 2019 in Iran as a part of the Ph.D.’s thesis of Health Policy Degree requirement by the S.SH (first author). Decision- and policy-makers, rehabilitation experts, faculty members, and practitioners (including Physiotherapists, Occupational therapists, and Orthotists/Prosthetists), were selected to participate in in-depth semi-structured interviews.

### Participants

Purposive and snowball sampling approaches were used to identify the participants. During the sampling, the research team tried to recruit individuals with a maximum variation in terms of specialty, employment status, gender, experience, and geographic location. The eligibility criteria for semi-structured interviews were to include: (1) health policy-makers and rehabilitation experts who had more than 3 years of executive experience in policy-making in regard to the physical rehabilitation services in Iran; (2) faculty members who had at least 3 years of experience in regard to clinical and theoretical education of physical rehabilitation; and finally, (3) practitioners who had at least 4 years of experience of providing physical rehabilitation services in practice, along with a valid registration number of the Medical Council of the Islamic Republic of Iran (IRIMC). The interview sessions continued until saturation was achieved, and no new information was generated. Two sessions with duplicate information were determined to confirm the data saturation.

### Data collection

In-depth semi-structured interviews were conducted by S.SH (a male Ph.D. Health Policy Candidate with health-related rehabilitation experiences) applying an interview guide including open-ended questions and suggested probes, developed based on the six-building block WHO framework (Table 1), which covers different dimensions of health system and provides a common language [24]. The primary questions were revised for further clarity based on the reflected feedbacks from initial interviews. Face-to-face interviews were conducted in big cities including Tehran, Shiraz, and Isfahan, and telephone and WhatsApp interviews were used for participants who lived in other cities or preferred it. Interviews were conducted in noiseless and favorable rooms, without any observer. The interviewer strived to ensure a high level of privacy to enable the participants to express their opinions freely. Besides two recorders were used for discussions, taking notes was done during the interview simultaneously. Immediately after each interview, recorded files were transcribed verbatim by S.SH, and then, the transcribed files were saved in Word Microsoft Office. All participants were allowed to assess their interview texts, and if required, they had the opportunity to amend it. The first author endeavored to provide the transcript up to 14 days after each interview. To secure the anonymity, a pseudonym was applied for naming the participants in initial texts and publications.

**Table 1:** Interview guide

**Questions**	
1. What are the effects of international sanctions and embargos on the provision of physical rehabilitation services (physiotherapy, occupational therapy, orthotics, and prosthetics) in Iran?	
*Probes:* How? Your experience?	
2. What are the effects of international sanctions and embargos on the financing of physical rehabilitation services (physiotherapy, occupational therapy, orthotics, and prosthetics) in Iran?	
*Probes*: How? Your experience?	
3. What are the effects of international sanctions and embargos on the governance and leadership of physical rehabilitation services (physiotherapy, occupational therapy, orthotics, and prosthetics) in Iran?	
*Probes*: How? Your experience?	
4. What are the effects of international sanctions and embargos on the essential equipment and devices of physical rehabilitation services (physiotherapy, occupational therapy, orthotics, and prosthetics) in Iran?	
*Probes*: How? Your experience?	
5. What are the effects of international sanctions and embargos on the education and human workforce of physical rehabilitation services (physiotherapy, occupational therapy, orthotics, and prosthetics) in Iran?	
*Probes*: How? Your experience?	
6. What are the effects of international sanctions and embargos on the information systems of physical rehabilitation services (physiotherapy, occupational therapy, orthotics, and prosthetics) in Iran?	
*Probes*: How? Your experience?	

To enhance the quality of reporting, the consolidated criteria for reporting qualitative research (COREQ) [25] and the standards for reporting qualitative research (SRQR) [26] were considered.

### Data analysis

**Fig. 1:**
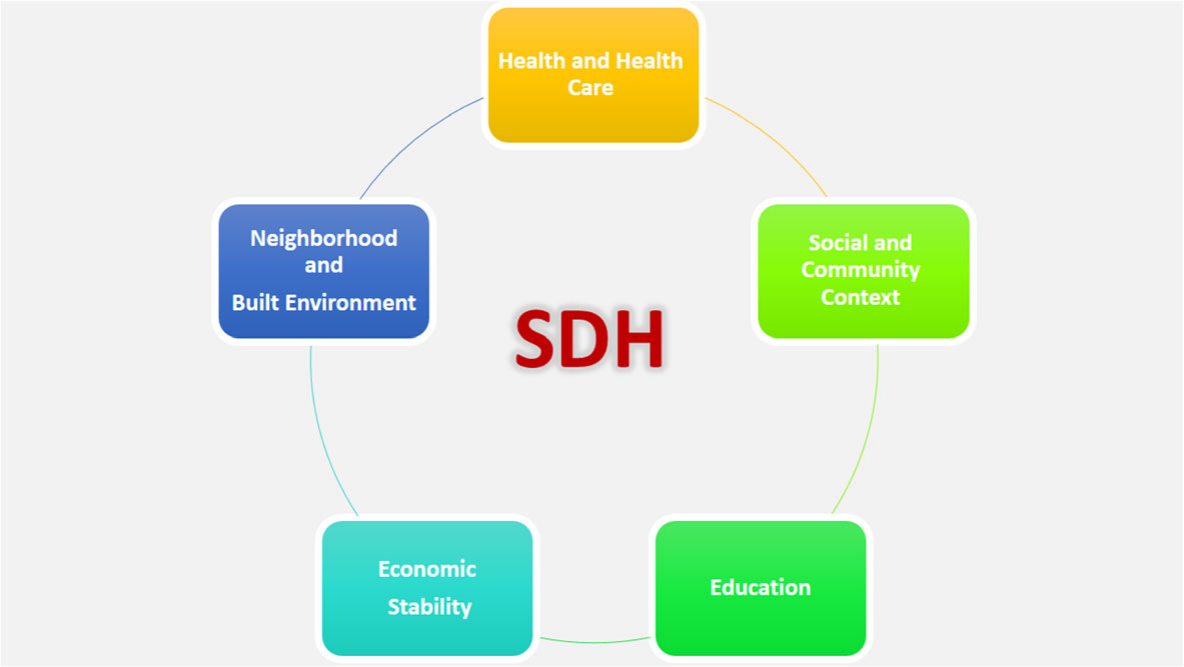
Social determinants of health framework (adapted from: Healthy People 2020: Social Determinants of Health)

Framework analysis approach was used to investigate the situation of the physical rehabilitation sector in Iran after the international sanctions as perceived by policy-makers, faculty members, and practitioners [27]. The analysis process was done along with the data collection process. Two of the authors (S.SH. and A.A.) read the transcripts several times independently. According to the framework analysis approach, the texts were divided into meaning units, and then, these identified units were condensed to develop codes and following sub-themes. At last, the research team discussed the sub-themes and also abstracted them to allocate the parent themes. Social Determinants of Health (SDH) framework, which has developed by Healthy People 2020 initiative, was applied to facilitate the analysis stages [28]. This framework consists of five constructs that can affect the health status and outcomes (Fig. 1). Disagreements among the authors were resolved through discussion.

Critical reflexivity was considered through data collection and analysis processes to diminish the potential risk of bias [29]. S.SH as the interviewer described his scientific and employment background at the beginning of each interview. Additionally, the involved authors in the analysis process had different scientific experiences that could reduce bias.

### Trustworthiness strategies

Based on the Lincoln and Guba framework, trustworthiness is the vital parameter for evaluating the rigor of qualitative findings. This framework includes dependability, credibility, confirmability, transferability, and authenticity [30]. In this study, several measures were adopted to improve the trustworthiness of findings as follows: stepwise replication in data analysis to improve the dependability, peer debriefing, prolonged immersion of the first author, and methods triangulation to improve the credibility, member-checking by authors and participants to improve the confirmability, and in-depth descriptions and purposive sampling to improve the transferability, and in final, considering citations from different participants to improve the authenticity.

### Ethical approval

All participants were invited to involve in the interviews through introductory WhatsApp and e-mail letter including the objectives and aims of the project. All participants were free to withdraw from the study at any stage. Each participant received a written information sheet in Persian and was asked to read, and then, sign the consent form. Additionally, consent was achieved verbally at the first of each interview session. Ethical approval was provided by the Research Ethics Committee of the School of Health Management and Information Sciences, Iran University of Medical Sciences, Tehran, Iran (IR/IUMS/REC/1397/889).

## Results

In total, 38 participants, including 11 health-policy makers, 7 rehabilitation experts, 8 faculty members, and 12 practitioners, involved in the study (Table 2). However, due to the employment status, a female practitioner declared not to participate in the study.

**Table 2:** Composition of participants

Participants	No.
Health policy-maker	11
Faculty member	8
Rehabilitation expert	7
Physiotherapist	5
Orthotist/Prosthetist	4
Occupational therapist	3

Through the interviews, participants pointed to a number of challenges facing the Iranian physical rehabilitation sector during the international sanctions period. The following explains the situation of this sector after the imposition of sanctions in accordance with the SDH framework in detail, and also, quotes from the in-depth interviews will be used for further clarity (Table 3).

**Table 3:** Themes, sub-themes, and suggested quotes

Themes	Sub-themes	Quotes
Socio-economic challenges	Inadequate funding	*“Much of the [Iranian] health and welfare system’s budget comes from oil and petrochemical sales. In recent years, government revenues have fallen sharply...” (Health policy-maker 3)*
	Rising inflation rate	*“So our costs have increased. We have to cover essential health services and not include any services like physiotherapy.” (Health policy-maker 6)*
	High unemployment rate	*“Our insurance funds are really in trouble. After the sanctions, many have become unemployed. They don’t even pay a premium!” (Health policy-maker 1)*
	Catastrophic expenditures	*“After the sanctions, prostheses have become really expensive. How can an amputee who often has no income pay around 120 million Rials [Iranian currency] for a below-knee prosthesis?” (Practitioner 9)*
	Inappropriate employment status of practitioners	*“Rental rates for a clinic have gone up. Many of my colleagues have closed down. Not really economical. The inflation of sanctions has destroyed us.” (Practitioner 10)*
		*“We have no job opportunities in other countries … But if there is some kind of international cooperation right now, the credibility of our degree will increase.” (Practitioner 4)*
Education challenges	Decreasing the international collaboration	*“Because of the sanctions, our department’s collaboration with other universities around the world has diminished. We used to exchange doctoral students. The students had very good experiences that were very valuable for the development of the field [occupational therapy].” (Faculty member 8)*
	Shortage of training devices and materials	*“After the sanctions, we do not have many advanced training tools” (Faculty member 3)*
International challenges	Rising challenges for patients from neighborhood countries	*“Many amputees came here from Afghanistan and received prostheses. But now that our raw materials are reduced, we can’t provide them with services.” (Rehabilitation expert 2)*
Service delivery challenges	Shortage of raw materials for producing the orthoses and prostheses	*“Although we work in a non-governmental organization, after the U.S. sanctions, there are not many devices. We used artificial foot, produced by a German company named Ottobock for below-knee amputees, the patients were very happy, but now we have to use Chinese low-quality feet. After a few days, the foot will break!” (Practitioner 4)*
	Hardening of the importing the needed equipment	*“We needed some devices like Shock wave therapy, but we couldn’t import them. Interestingly, some equipment is only available in large cities.” (Practitioner 9)*
	Inappropriate infrastructures	*“The rehabilitation infrastructures are really worn out, especially in the government sector. The facilities of the State Welfare Organization were from twenty to thirty years ago. Many facilities and features have not been updated. These sanctions have greatly restricted our works.” (Rehabilitation expert 4)*
		*“Many of our tools are outdated. For example, we were not given the technology of new orthoses. We are still building an orthosis that has been around for the past twenty years.” (Practitioner 8)*
	Impossibility to use external assistance	*“Many international organizations, such as the World Health Organization, the United Nations, and others, allocate large funds annually for the services of disadvantaged groups such as the disabled and the elderly, but because we are sanctioned, these resources are not allocated to us …” (Rehabilitation expert 5)*
		*“We tried to use the services of international accreditation agencies to improve the quality of services, but they said that you are sanctioned and we are not going to cooperate with you ...” (Rehabilitation expert 1)*

### Socio-economic challenges

The findings demonstrated that the international sanctions reduced the Iranian oil’s export and revenues, as the main source of financing of health care and wellbeing services significantly. Many health decision- and policy-makers suggested that there are no sufficient funds for effective financing of physical rehabilitation services following the harsh sanctions. For example, one policy-maker stated:

*"Much of the [Iranian] health and welfare system's budget comes from oil and petrochemical sales. In recent years, government revenues have fallen sharply. The government is trying to divide existing financial resources among different sectors. It is natural not to devote specific [financial] resources for the rehabilitation!"(Health policy-maker 3)*

Many participants identified the sharp rising inflation rate as the other negative impact of international sanctions, which can put various barriers in the way of optimal financing. The results showed that as inflation increased, health insurers’ expenditures increased sharply, so some insurance companies were struggling to remove some services such as rehabilitation from their list of covered services.

*"So our costs have increased. We have to cover essential health services and not include any services like physiotherapy."(Health policy-maker 6)*

Increased unemployment rate is another factor that directly and indirectly affects the financing of the health system, including rehabilitation services. The high unemployment rate has dramatically reduced the premiums collected by health insurers.

*"Our insurance funds are really in trouble. After the sanctions, many have become unemployed. They don't even pay a premium!"(Health policy-maker 1)*

In addition, the majority of providers described that following the recent sanctions and ad hoc economic pressures, the reimbursement period has been very long. Therefore, providers had to consider the patient direct payments as the main source.

*"Insurance has not yet paid last year's invoice. Well ... how do I cover the costs? I have to receive out-of-pocket (OOP) payments." (Practitioner12)*

These direct payments have confronted many households with catastrophic expenditures and led some households to go below the poverty line. Indeed, the high cost of rehabilitation services to recipients, and since many recipients are chronically disabled and also come from low-income families, the economic burden is multiplied.

*"After the sanctions, prostheses have become really expensive. How can an amputee who often has no income pay around 120 million Rials [Iranian currency] for a below-knee prosthesis?" (Practitioner 9)*

On the other hand, the harsh economic situation following the imposition of the sanctions has affected the employment status of physiotherapy, occupational therapy, and orthotics and prosthetics graduates. Practitioners demonstrated that following the sanctions, government employment has declined significantly and private employment has been very difficult due to inflation and rising costs, thus, many private rehabilitation centers closed in recent months.

*"Rental rates for a clinic have gone up. Many of my colleagues have closed down. Not really economical. The inflation of sanctions has destroyed us." (Practitioner 10)*

The declined strength of Iranian non-governmental organizations (NGOs) and charities following the devaluation of the national currency and declined in economic growth, was another challenge identified by the policy-makers that has encountered many vulnerable groups with considerable obstacles.

*"Many rehabilitation services were provided to disabled people through charities and disease associations. But in recent years, because of the poor economic situation, the resources of these institutions have also declined."(Faculty member 7)*

### Education challenges

Based on the findings of the study, academic education of physical rehabilitation disciplines (such as physiotherapy, occupational therapy, orthotics and prosthetics) has been strongly affected after international sanctions, which can curtail the scientific progress in this domain. Rejection of relevant articles by some journals, inability to attend the international scientific meetings and paying the registration fee, decreasing the international collaboration among relevant faculties and student exchange, and also lack of access to international training courses, were the most common challenges expressed.

*"Because of the sanctions, our department's collaboration with other universities around the world has diminished. We used to exchange doctoral students. The students had very good experiences that were very valuable for the development of the field [occupational therapy]." (Faculty member 8)*

Many participants also perceived challenges with regard to the physical rehabilitation human resources after the sanctions. The shortage of training devices and materials and also the lack of sufficient funds for clinical courses were identified.

*"The budget for our department has declined sharply. Therefore, we are not able to provide the necessary equipment. The result is a decline in the quality of graduates." (Faculty member 2)*

### International challenges

These sanctions and embargos had also negative effects on patients from other countries who received rehabilitation services such as orthoses and prostheses in Iran. Due to the war and internal conflicts in many parts of the Middle East region, demands for physical rehabilitation services are rising. Therefore, some patients come to Iran for rehabilitation services, especially for artificial limbs. However, following the imposition of new sanctions, and problems arisen, these patients have faced serious challenges.

*"Many amputees came here from Afghanistan and received prostheses. But now that our raw materials are reduced, we can't provide them with services."(Rehabilitation expert 2)*

### Service delivery challenges

Regarding the delivery of physical rehabilitation services, several challenges were described by participants, such as the shortage of raw materials for producing the orthoses and prostheses. A Prosthetist who worked in Iranian Red Crescent Society, criticized the U.S. sanctions and mentioned the shortage of crucial materials for producing the optimal prostheses.

*"Although we work in a non-governmental organization, after the U.S. sanctions, there are not many devices. We used artificial foot, produced by a German company named Ottobock for below-knee amputees, the patients were very happy, but now we have to use Chinese low-quality feet. After a few days, the foot will break!" (Practitioner 4)*

Participants pointed to the difficult, costly, and lengthy process of importing the needed equipment, due to the banned bank transaction following the international suctions against Iran. They believed that this situation has made many of the equipment needed to provide services unavailable, especially in outlying areas*.*

*"We needed some devices like Shock wave therapy, but we couldn't import them. Interestingly, some equipment is only available in large cities." (Practitioner 9)*

A number of policy-makers expressed that the sanctions have reduced the Iranian health sector’s capacities resulting in the neglect of physical rehabilitation services. In addition, a rehabilitation expert believed that since the health sector infrastructures are not well developed, the main proportion of health care services is not provided, especially rehabilitation interventions which need governmental supports.

*"The rehabilitation infrastructures are really worn out, especially in the government sector. The facilities of the State Welfare Organization were from twenty to thirty years ago. Many facilities and features have not been updated. These sanctions have greatly restricted our works."(Rehabilitation expert 4)*

Furthermore, corrupt activities in the provision of services were other mentioned pitfalls. Through the interviews, participants described corruption as one of the outcomes of sanctions. They believed that the imposed limitations have led to abuse by some providers. One university professor expressed it as follows:

*"The shortage of some equipment has incited the clinics to sour the prices as much as they desire." (Faculty member 2)*

A major proportion of available devices and equipment are outdated. Therefore, the quality of interventions has been reduced in recent years. One Orthotist reported this as follows:

*"Many of our tools are outdated. For example, we were not given the technology of new orthoses. We are still building an orthosis that has been around for the past twenty years." (Practitioner 8)*

The use of external assistance is one of the common ways of providing rehabilitation services to disabled people, especially in developing countries. Nonetheless, Iran’s rehabilitation sector cannot benefit from these opportunities because of the banking restrictions.

*"Many international organizations, such as the World Health Organization, the United Nations, and others, allocate large funds annually for the services of disadvantaged groups such as the disabled and the elderly, but because we are sanctioned, these resources are not allocated to us … "(Rehabilitation expert 5)*

In addition, the declined strength of Iranian non-governmental organizations (NGOs) and charities following the devaluation of the national currency and declined in economic growth, was another challenge identified by the policy-makers that has encountered many vulnerable groups with considerable obstacles.

*"Many rehabilitation services were provided to disabled people through charities and disease associations. But in recent years, because of the poor economic situation, the resources of these institutions have also declined."(Faculty member 7)*

Rehabilitation experts expressed that failure to use the foreign accredited institutions is another negative impact of international sanctions on the governance of physical rehabilitation services in Iran. Indeed, almost all of the accreditation institutes such as the Commission on Accreditation of Rehabilitation Facilities (CARF), are not allowed to cooperate with the Iranian physical rehabilitation sector.

*"We tried to use the services of international accreditation agencies to improve the quality of services, but they said that you are sanctioned and we are not going to cooperate with you ..."(Rehabilitation expert 1)*

Furthermore, due to sanctions and lack of reputable dealers, many of the imported equipment are also sold in cash in Iran. However, in many countries around the world, this equipment is provided with long-term installments by reputable dealers.

*"Rehabilitation equipment is expensive. On the other hand, after the sanctions, the dealerships are out of the country and we have to buy the equipment in cash … " (Faculty member 7)*

Lack of a valid warranty for equipment and devices is another challenge. Many practitioners believed that there are not adequate instructions on how to use the device. Also, it can be very difficult to repair when the device crashes.

*"There are a lot of things [devices] in our center right now that are broken. No one knows how to repair them. The dealership that we bought from them has also closed." (Practitioner 11)*

In despite these, during the interviews, the participants stated that the development of Knowledge enterprises and also the use of reverse engineering approaches has provided opportunities for domestic production of the required devices and technologies.

*"Recently, with the development of knowledge-based companies, some devices have been developed by experts in these companies." (Rehabilitation expert 2)*

Many policy-makers mentioned the shortage of comprehensive monitoring services to follow-up the interventions’ effects. In fact, since international sanctions have banned the import of modern technologies, the physical rehabilitation sector information system is affected.

*"There are many good tools for monitoring and evaluating interventions, but we cannot import because we are sanctions."(Health policy-maker 6)*

Although using telerehabilitation platforms for providing the physical rehabilitation services in remote areas has been considered in recent years, the lack of relevant equipment and technologies have prevented applying these provision approaches.

*"We would have liked to use telerehabilitation and telemedicine for remote areas, but [relevant experts] said the equipment needed was imported and could not be accessed after the boycott."(Health policy-maker 11)*

## Discussion

This study is the first study to investigate the situation of Iranian physical rehabilitation sector after the imposition of international sanctions. The findings would suggest that the sanctions and embargoes had significant adverse effects on physical rehabilitation sector. Participants expressed a number of challenges of imposed sanctions in terms of main dimensions of SDH framework.

Financing is another functions of Iranian physical rehabilitation sector which was affected meaningfully. Based on the evidence, Iran’s gross domestic product (GDP) and also economic growth rate have reduced sharply, which led to a range of limitations in health and welfare funding [2]. In accordance with the government’s reports, only one-sixth of the Disability Protection Act budget has been allocated. Therefore, OOP payments are the common mechanism of payment for health care services, especially physical rehabilitation [31]. As a result, a significant proportion of households has confronted with CEs and also refuse to receive services [7]. In addition, policy-makers and providers demonstrated the negative impacts of high rate of inflation on the price of raw materials and equipment as well as rental rates. The other studies also have reported the adverse impacts of sanction-borne inflation on health sector and its sub-systems funding [21, 32]. As a whole, disabled and chronically ill patients who make up a large portion of physical rehabilitation recipients, are most affected following economic crisis. For instance, after the economic recession, vulnerable groups had limited access to health care services in Greece, Honduras, Iraq, and Syria [12, 33–36]. These findings are in agreement with a UNICEF statement which reported that most vulnerable and least culpable groups endure the heaviest consequences [37].

Furthermore, academic education of physical rehabilitation disciplines was another main domain affected during the international sanctions. Although the World Medical Association (WMA) rejects any academic sanction [38], limitations against scientific articles, attending scientific meetings, and international collaborations were commonly identified challenges through interviews. A recent publication concluded that the academic boycotts and sanctions can curtail the researchers’ progress and freedom [21]. Finally, since the international cooperation was reduced in recent years, information and accreditation systems have not developed in physical rehabilitation sector. Therefore, in some cases, the interventions have not sufficient quality [39]. This problem has been observed in Iranian medicine sector, which many international companies are refusing to cooperate and supply medicine to Iran [40, 41].

The shortage of raw materials and equipment was identified as the main challenge during the sanctions. Since imports had fallen sharply, the imported materials were scarcely available [23]. Further, the prices of raw materials and equipment raised considerably, sometimes doubled or tripled. In response to this situation, using low-quality materials produced in Iran or imported from eastern countries, became the common strategy [7, 19]. Therefore, a major proportion of clients such as amputees has been deprived of equitable and safe access to services. More importantly, through the interview sessions, the consequences of sanctions along with the poor management were considered to be potentially related to corruption in the provision of physical rehabilitation services, especially, physiotherapy and orthotics/prosthetics. A recent study also reported that corrupt activities are more prevalent in sanctioned nations [42]. Another notable finding was the adverse impacts of sanctions on patients from neighboring countries. Regarding the war and internal conflicts in Middle-East region, the demand for physical rehabilitation services has increased during the past decades [43]. Consequently, a large number of injured people from Afghanistan, Iraq, Syria and Yemen, come to Iran to receive rehabilitation services. However, in recent years and after the sanctions were tightened, they have faced serious problems [44].

### Limitations

Our study has several limitations; firstly, only one author (SSH) conducted the interviews, which can have influenced the quality of raw data. Secondly, the data was restricted to semi-structured interviews and would gain more advantage from a quantitative survey. Thirdly, based on the predefined protocol, patients/clients were not included in this study, who could provide good information. Lastly, since the sanctions regimes are international macro policies, it is difficult to draw a clear straightforward cause and effect relationship with regard to their effects on the studied field, and only a description of the situation was presented.

## Conclusions

International sanctions and embargos had considerable multifaceted effects on the Iranian physical rehabilitation sector. Possibly, disabled and vulnerable groups who usually belong to the poor population, have confronted with harsh barriers to access the physical rehabilitation services. In addition, the current sanctions against Iran had also negative impacts on the utilization of these services by relevant patients who come from neighboring countries to receive them. Therefore, the international community must be aware of these effects and be concerned about their irreparable consequences, and work to end them.

## Data Availability

The data collected and analyzed during the study are available from the corresponding author on reasonable request.

## Abbreviations

JCPOA: Joint Comprehensive Plan of Action
UN: United Nations
WHO: World Health Organization
COREQ: Consolidated criteria for reporting qualitative research
SRQR: Standards for reporting qualitative research
CEs: Catastrophic expenditures
NGOs: Non-governmental organizations
CARF: Commission on Accreditation of Rehabilitation Facilities
GDP: Gross domestic product
OOP: Out-of-pocket
